# MWA Versus RFA for Perivascular and Peribiliary CRLM: A Retrospective Patient- and Lesion-Based Analysis of Two Historical Cohorts

**DOI:** 10.1007/s00270-016-1413-3

**Published:** 2016-07-07

**Authors:** Aukje A. J. M. van Tilborg, Hester J. Scheffer, Marcus C. de Jong, Laurien G. P. H. Vroomen, Karin Nielsen, Cornelis van Kuijk, Petrousjka M. P. van den Tol, Martijn R. Meijerink

**Affiliations:** 1 Department of Radiology and Nuclear Medicine, VU University Medical Centre, De Boelelaan 1117, 1081 Amsterdam, The Netherlands; 2 Department of Surgical Oncology, VU University Medical Centre, Amsterdam, The Netherlands

**Keywords:** Radiofrequency, Microwave, Ablation, Colorectal liver metastases, Peribiliary, Perivascular

## Abstract

**Purpose:**

To retrospectively analyse the safety and efficacy of radiofrequency ablation (RFA) versus microwave ablation (MWA) in the treatment of unresectable colorectal liver metastases (CRLM) in proximity to large vessels and/or major bile ducts.

**Method and Materials:**

A database search was performed to include patients with unresectable histologically proven and/or ^18^F–FDG–PET avid CRLM who were treated with RFA or MWA between January 2001 and September 2014 in a single centre. All lesions that were considered to have a peribiliary and/or perivascular location were included. Univariate logistic regression analysis was performed to assess the distribution of patient, tumour and procedure characteristics. Multivariate logistic regression was used to correct for potential confounders.

**Results:**

Two hundred and forty-three patients with 774 unresectable CRLM were ablated. One hundred and twenty-two patients (78 males; 44 females) had at least one perivascular or peribiliary lesion (*n* = 199). Primary efficacy rate of RFA was superior to MWA after 3 and 12 months of follow-up (*P* = 0.010 and *P* = 0.022); however, after multivariate analysis this difference was non-significant at 12 months (*P* = 0.078) and vanished after repeat ablations (*P* = 0.39). More CTCAE grade III complications occurred after MWA versus RFA (18.8 vs. 7.9 %; *P* = 0.094); biliary complications were especially common after peribiliary MWA (*P* = 0.002).

**Conclusion:**

For perivascular CRLM, RFA and MWA are both safe treatment options that appear equally effective. For peribiliary CRLM, MWA has a higher complication rate than RFA, with similar efficacy. Based on these results, it is advised to use RFA for lesions in the proximity of major bile ducts.

## Introduction

Colorectal cancer is the third most common malignancy worldwide and the second most common cause of cancer death in developed countries [1]. Approximately 50 % of patients develop colorectal liver metastases (CRLM), yet only a minority (10–15 %) is feasible for hepatic resection. Five-year survival after liver resection ranges between 31 and 58 % in carefully selected patients [2, 3]. Thermal tumour ablation, especially radiofrequency (RFA) and microwave ablation (MWA), is commonly employed and widely available. Five-year survival following RFA varies between 17 and 51 % [4]. The long-term results of RFA are well reported and demonstrate an excellent safety profile and good primary efficacy rate and assisted efficacy rate for small CRLM [5–7]. RFA is considered less suitable for lesions in close proximity to large vessels because of the so-called ‘heat-sink’ effect, where heat is carried away by the flowing blood, leading to higher local site recurrence rates. MWA does not rely on the passive conduction of heat and therefore is often preferred over RFA for perivascular CRLM [8, 9]. However, microwave systems also face several limitations including shaft heating, large diameter probes, less predictable ablation zones, and higher peak temperatures with the potential hazard of occluding important vessels or damaging vital structures such as the major bile ducts [8, 10].

The primary aim of this study was to retrospectively analyse the safety and efficacy of RFA versus MWA in the treatment of unresectable CRLM in proximity to large vessels and/or major bile ducts.

## Materials and Methods

### Patient Selection (Fig. 1)

A retrospective comparative analysis of all patients with histologically proven and/or fluorine-18 (^18^F) fluorodeoxyglucose (FDG) positron emission tomography (PET) avid CRLM who underwent either RFA or MWA with or without additional resection was performed. Data from patients treated between January 2001 and September 2014 were extracted from a prospectively maintained registry database. From 2007 onwards, the institution started using MWA for perivascular lesions. All demographic, clinical, operative, pathological, and follow-up data were collected. Patients with missing data or patients lost to follow-up (follow-up <12 months after ablation) were excluded, as were patients in whom a contrast-enhanced CT or magnetic resonance imaging (MRI) acquired maximum 10 weeks prior to the initial procedure was unavailable. An experienced reviewer, blinded to the final approach and outcome, included all lesions that were considered perivascular and/or peribiliary. Perivascular lesions were defined as lesions with its nearest margin ≤5 mm from a vessel of at least 4 mm in diameter; peribiliary lesions were situated ≤5 mm to the common hepatic duct, main right or left hepatic duct. Patients without perivascular and/or peribiliary lesions were excluded from the analysis as well as patients in whom all perivascular lesions were resected. Lesions treated with thermal ablation that were undetectable on pre-procedural CT but found and treated during laparotomy were also excluded from analysis. The medical history, including all pre- and post-procedural imaging, of all included patients was evaluated using an electronic database search [11]. The follow-up imaging protocol consisted of 3, 6, 9, 12, 18 and 24 months of follow-up CT scans and 6, 12 and 24 months of follow-up ^18^F–FDG–PET scans followed by annual PET and CT scans, if no recurrence was present. Complications were graded according to the common terminology criteria of adverse events (CTCAE version 4.0) and divided into three causal categories: (1) electrode or antenna placement, (2) thermal injury and (3) secondary to the general procedure [12]. Efficacy was assessed according to the standardization of terminology and reporting criteria [13]. The primary efficacy rate was defined as the percentage of lesions who had no sign for local recurrence after a follow-up period of 3 and 12 months after the initial procedure; the assisted efficacy rate was defined as the percentage of lesions with no sign for recurrence at least 12 months after the last procedure—including locally recurring lesions that were retreated, regardless of the technique used. Patient characteristics, tumour burden, procedural characteristics and treatment characteristics were assessed to detect potential confounders. All procedures were performed according to the guidelines for good clinical practice (GCP). Patients consented to the anonymized registration of relevant medical information in the registry database. For the retrospective analysis of these data, formal review board approval was waived since the patients were not subjected to procedural or behavioural rules.

**Fig. 1:**
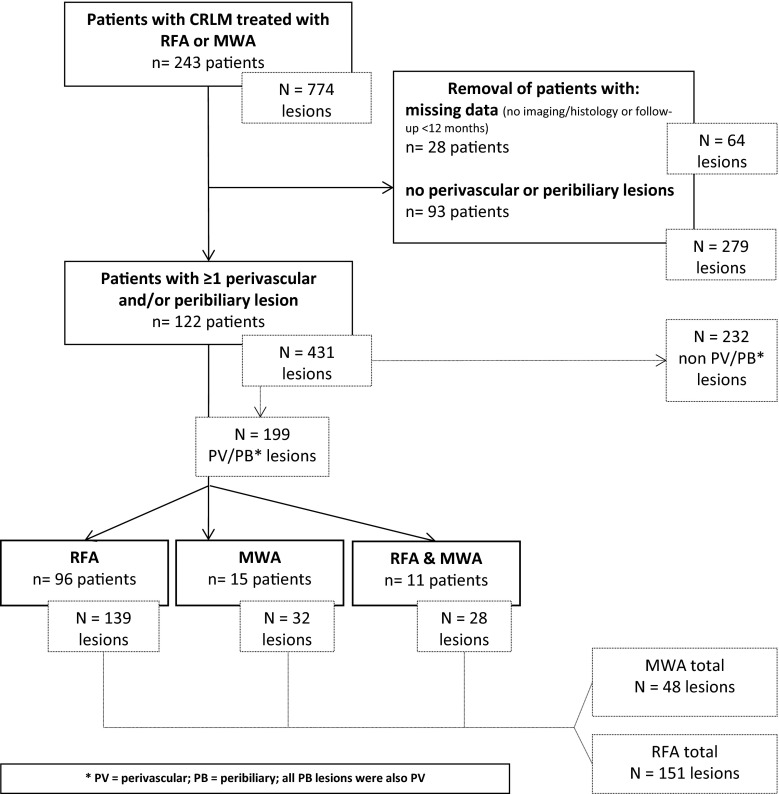
Flowchart of patient and lesion selection

### RFA and MWA Procedures

All patients were discussed in our hepatobiliary multidisciplinary tumour board. Criteria for unresectability of CRLM were major liver vascular involvement (e.g. of all three hepatic veins, the portal vein bifurcation or the retrohepatic vena cava), bilateral dissemination requiring liver resection that would result in inadequate future liver remnant, substantial and relevant co-morbidities, and an impaired general health status. Lesions in direct contact with the main bile ducts were considered unsuitable for thermal ablation. Before 2007, MWA was not available in our centre and all lesions were treated with RFA. From 2007 onwards, the choice between RFA and MWA was based on operator preference. In general, MWA was preferred for lesions in the vicinity of large blood vessels because of the alleged lower incidence of heat-sink-induced recurrences and RFA for lesions in the vicinity of the biliary tract, the diaphragm or the intestine because of the presumed superior ablation-zone predictability. In two patients, both treated with RFA, a so-called Pringle manoeuvre was performed, placing a large haemostat to temporarily interrupt the flow of blood through both the hepatic artery and the portal vein. All procedures were performed according to the manufacturer’s protocol in consensus with the cardiovascular and interventional radiological society of Europe quality improvement guidelines [14]. For RFA, the 2.0- to5.0-cm expandable needle electrodes were used in combination with the RF 3000 generator (LeVeen, Boston Scientific, USA). For MWA, we used 3.7-cm microwave antenna(s) (Evident, Covidien, Dublin, Ireland). The primary endpoint for a technically successful ablation was a fully hyperechoic ablation zone including a tumour-free margin of at least one centimetre on IOUS. For larger or non-spherical lesions, the electrodes or antennas were repositioned for one or more overlapping ablations whenever considered necessary. When employing MWA for larger lesions, up to three antennas were simultaneously used to enhance the ablation zone conferring to manufacturer’s protocol. In general, an open approach was favoured for the initial procedure. For new or recurring unresectable lesions in patients who already underwent open ablation and/or resection, the percutaneous approach was preferred if all lesions were suitable for the percutaneous approach, depending on size, location and visibility with CT or transabdominal ultrasound.

### Data Management and Statistical Analysis

We used univariate logistic regression analysis to evaluate the distribution of variables. To assess subject variables [age, sex, Eastern Cooperative Oncology Group (ECOG) performance status, primary tumour site (rectum/colon), origin of CRLM (synchronous/metachronous), pre- or post-procedural chemotherapy] and survival characteristics, patients were divided into one of three groups: RFA alone, MWA alone and RFA plus MWA (patients who had retreatments using the alternate technique). To assess procedure (approach, complications) and lesion characteristics (size, anatomical and perivascular or peribiliary location, 3- and 12-month primary and assisted efficacy rates), we assigned every lesion and every procedures to either RFA or MWA. Recurring lesions retreated using the alternate technique were classified according to the initial treatment. Multivariate logistic regression was used to assess significant variables in univariate analysis to correct for potential confounding. Any variables with a *P* < 0.15 in univariate analysis were entered into a multivariate model. The Kaplan–Meier method was used for survival analysis using the x^2^ log-rank analysis to test equality of survival distributions between the three treatment groups: RFA, MWA and both. Final statistical results were considered significant if *P* < 0.05. For statistical analysis, SPSS software version 20.0 for windows (IBM, Armonk, NY, USA) was used.

## Results

### Patient, Lesion and Procedure Characteristics (Table 1)

Patient, lesion and procedure characteristics are listed in Table 1. A total of 199 lesions in 122 patients were located perivascular and/or peribiliary. No lesions were located peribiliary alone, 161 lesions had a perivascular location alone and 38 lesions were located both peribiliary and perivascular. From the 38 peribiliary lesions, 31 were treated with RFA and 7 with MWA (*P* = 0.36). Mean size of ablated CRLM was 2.4 cm (range 0.2–6.4 cm), with no significant difference between the RFA and MWA group (2.4 vs. 2.5 cm, *P* = 0.72). Of the 199 lesions treated with RFA/MWA, 186 were treated during open laparotomy and 13 were approached percutaneously (*P* = 0.0007). Resection of CRLM in the same session was performed in 67 patients. All RFA and MWA procedures were considered technically successful. Chemotherapy regimens were heterogeneous and susceptible to changes in insight over the past 15 years, which rendered subgroup analysis difficult. Nevertheless, a similar percentage of patients in both groups received chemotherapy at some time during the course of the disease (*P* = 0.557).

**Table 1:** Logistic regression analysis (univariate)—technique versus patient, lesion and technique characteristics (*n* = 122 patients)

Patient characteristics (*n* = 122 patients)	RFA alone	MWA alone	RFA & MWA	Odds ratio (95 % CI)	*P* value
Age [in years; mean (range)]	61 (35–78)	63 (26–81)	65 (56–74)	1.008 (0.957–1.062)	0.764
Sex (male/female)	60/36 (96)	12/3 (15)	6/5 (11)	0.500 (0.152–1.640)	0.253
ECOG performance status (0/1/2)	87/7/2	13/1/1	10/1/0	1.484 (0.554–3.976)	0.484
Primary (rectum/colon)	36/60 (96)	7/8 (15)	4/7 (11)	0.692 (0.247–1.940)	0.484
Origin (synchronous/metachronous)	40/56 (96)	4/11 (15)	5/6 (11)	2.344 (0.716–7.674)	0.159
Chemotherapy (no/yes)	22/74 (96)	6/9 (15)	3/8 (11)	0.711 (0.228–2.220)	0.557

Lesion characteristics (*n* = 199 lesions)	RFA	MWA	Odds ratio (95 % CI)	*P* value
Size [mm; mean (range)]	24 (2–68)	25 (range 4–65)	1.004 (0.982–1.026)	0.72
Anatomical segment (segment I–VIII)	15/8/5/26/20/9/20/48	1/4/1/6/5/5/11/15	0.21–1.78 (0.03–6.13)	0.16 – 0.70
Location (perivasc/peribil/both)	120/0/31 (151)	41/0/7 (48)	1.513 (0.619–3.698)	0.36

Technique characteristics (*n* = 199 lesions)	RFA	MWA	Odds ratio (95 % CI)	*P* value
Approach (open/perc)	147/4 (151)	39/9 (48)	8.481 (2.480–29.002)	0.0007

*P value for difference between RFA alone and MWA alone groups; RFA and MWA group not included in analysis

### Primary and Assisted Efficacy Rates (Tables 2, 3)

At 3 and 12 months, local ablation site recurrence was 9.3 % (14/151) and 21.9 % (33/151) for RFA treated lesions versus 25.0 % (12/48) and 39.6 % (19/48) for MWA-treated lesions (*P* = 0.010 and *P* = 0.022). In the RFA group, repeat procedures eventually controlled 45 % (15/33) of the recurring lesions using re-RFA (*n* = 9), MWA (*n* = 3), resection (*n* = 2) and stereotactic radiotherapy (*n* = 1). For the MWA group, repeat procedures were successful in 52 % (10/19) using re-MWA (*n* = 5), RFA (*n* = 3) and resection (*n* = 2). Therefore, 11.9 % (18/151) of initially RF-treated lesions versus 18.8 % (9/48) of initially MW-treated lesions were not locally controlled; this difference was not statistically significant (*P* = 0.13). Local site recurrence for the percutaneous procedure was 25 % (1/4) in the RFA group and 44 % (4/9) in the MWA group. Assessment of all possible confounders in a multivariate analysis revealed no significant difference between RFA and MWA in outcome after 12 months and after repeat procedures (*P* = 0.078 and *P* = 0.39). The only two parameters significantly associated with outcome in terms of primary and assisted efficacy rate were lesion size (*P* = 0.00003–0.011) and approach (*P* = 0.015–0.843). For perivascular/peribiliary CRLM <3, 3–5 and >5 cm efficacy rate was, respectively, 93.2, 80.0 and 64 % after 3 months; 85.0, 68.0 and 35.7 % after 12 months and 90.4, 78.2 and 50.0 % after repeat procedures.

**Table 2:** Logistic regression analysis (uni- and multivariate)—technique versus outcome (*n* = 199 lesions)

	RFA	MWA	Odds ratio (95 % CI)	*P* value
Outcome per lesion (*n* = 199)				
* Univariate analysis*				
Primary efficacy rate (3 months)	136/151 (90.1 %)	36/48 (75.0 %)	0.331 (0.142–0.769)	*0.010*
Primary efficacy rate (12 months)	118/151 (78.1 %)	29/48 (60.4 %)	0.444 (0.222–0.887)	*0.022*
Assisted efficacy rate	133/151 (88.1 %)	39/48 (81.3 %)	0.514 (0.219–1.207)	0.13
* Multivariate analysis*				
Primary efficacy rate (3 months)	–	–	0.311 (0.130–0.746)	0.0088
Primary efficacy rate (12 months)	–	–	0.520 (0.251–1.076)	0.078
Assisted efficacy rate	–	–	0.669 (0.266–1.683)	0.39

**Table 3:** Logistic regression analysis (univariate)—outcome versus lesion characteristics

	*P* value—PTE 3 months	*P* value—PTE 12 months	*P* value—ATE
Lesion characteristics (*n* = 199 lesions)			
Size [mm; mean (range)]	0.001	0.000033	0.011
Anatomical segment (segment I–VIII)	0.01–0.999	0.066–0.736	0.27–0.999
Approach (open)	0.843	0.029	0.015

### Survival (Fig. 2)

Median overall survival (OS) was 63.0 months (95 % CI 45.3–80.7) from primary tumour diagnosis and 42.0 months (95 % CI 36.7–47.3) from the first ablation procedure for the entire group. Median overall survival was not reached for the MWA group after a mean follow-up period of 49 months from primary tumour diagnosis and 31 months from the perivascular/peribiliary ablation. Survival distributions between the group of patients that underwent RFA alone, MWA alone or both treatments were not statistically different for both the survival times from primary tumour diagnosis (x^2^ = 0.215; *P* = 0.898) and survival times from ablation (x^2^ = 1.161; *P* = 0.559).

**Fig. 2:**
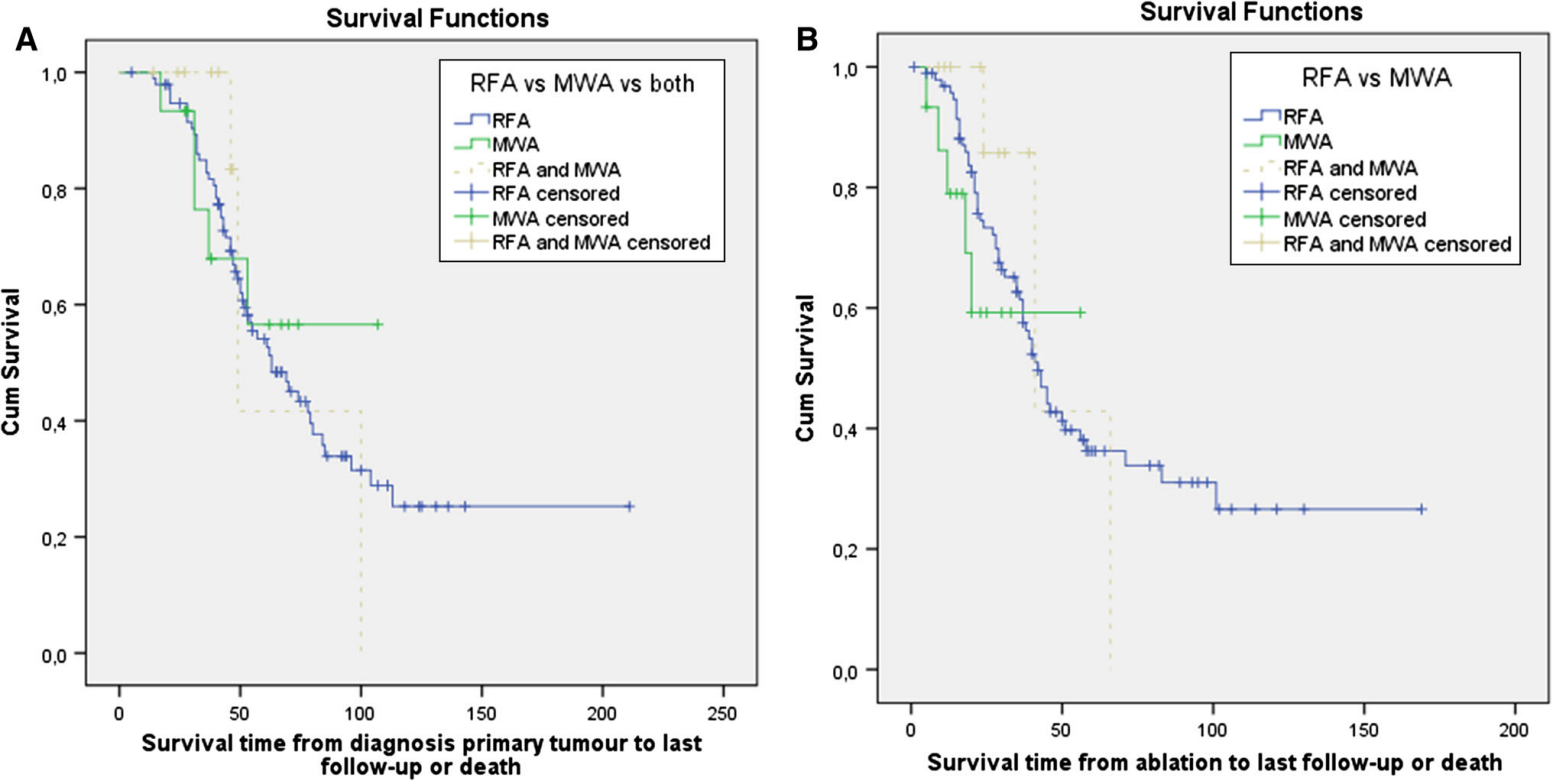
Kaplan–Meier curves showing overall survival from primary tumour diagnosis and from the first ablation procedure. Patients were distributed to the RFA group, MWA group or RFA plus MWA group based on the specific ablation procedures they had undergone. Survival distributions were not statistically different between the treatment groups for the survival times from primary tumour diagnosis (x^2^ = 0.215; *P* = 0.898) and from ablation (x^2^ = 1.161; *P* = 0.559)

### Complications (Table 4)

There were no direct procedure-related mortalities. Although not reaching significance (*P* = 0.094), there were more CTCAE grade III complications in the MWA group 18.8 % (6/32) compared to the RFA group 7.9 % (11/140). Biliary complications (biloma/biliary leakage, biliary obstruction, bilio-pleural fistula) were especially common after peribiliary MWA 57.1 % (4/7) versus RFA 3.2 % (1/31) reaching significance (*P* = 0.002). For both techniques, the number of complications did not decrease with operator experience. In the smaller MWA group, we saw five complications (two grade III) for the first 50 % of procedures versus seven complications (four grade III) for the second 50 % of procedures. For the first versus the second, 50 % of RFA procedures, respectively, 13 (five grade III) versus 16 (six grade III) complications were registered.

**Table 4:** Complications—RFA versus MWA (total 172 procedures: 140 RFA; 32 MWA; *n* = 122 patients)

	CTCAE grade I/II (*n* = 23 patients)	RFA	MWA	Treatment
		17/140 (12.1 %)	6/32 (18.8 %)	*P* = 0.39
Probe injury	Pneumothorax	1	1	Conservative
Thermal injury	Fever	2	1	No
	Nausea	2	0	No
	Pain	4	0	NSAIDS
	Pain and fever	1	1	NSAIDS
Related to general procedure	Urinary tract infection	0	1	Antibiotics
	Dysregulated diabetes mellitus	1	0	Insulin
	Plexus brachialis neuralgia	0	1	Neurology consult & physiotherapy
	Pneumonia	3	0	Antibiotics
	Transient neurological disorder	2	0	Neurology consult
	Grounding pad skin burn	1	–	Antibiotic cream
	Benign cardiac arrhythmia	0	1	No
	CTCAE grade III (*n* = 17 patients)	11/140 (7.9 %)	6/32 (18.8 %)	*P* = 0.094
Probe injury	Hepatic haemorrhage	2	1	Blood transfusion (2); coiling (1)
Thermal injury	Subphrenical abscess	0	1	Drainage
	Liver abscess	6	0	Drainage
	Biloma/biliary leakage	1	2	Drainage
	Biliary obstruction	0	1	PTCD with stent placement
	Bilio-pleural fistula	0	1	Pleural drain & biliary stent for flow diversion
Related to general procedure	Pulmonary embolism	1	0	Heparin i.v.
	Bacteremia needing antibiotics	1	0	Antibiotics

## Discussion

There is surprisingly little literature comparing RFA with MWA for CRLM. There are no series available that make a direct comparison between the two techniques. Although local site recurrence rates and established survival outcomes after RFA or MWA seem similar, apparent inclusion and exclusion biases make it difficult to perform a fair meta-analysis. In the treatment of hepatocellular carcinoma, the vast majority of studies showed either an equivalent role for both techniques or an upper hand for MWA [15–23].

In RFA, an alternating electrical circuit is created through the body to conduct RF current. Because of the abundance of ionic fluid present, RF current is able to pass through tissue. However, as tissue is not a perfect conductor, the current causes resistive heating (the Joule effect). MWA represents a specific form of dielectric heating, where the dielectric material is tissue. Dielectric heating occurs when an alternating electromagnetic (EM) field is applied to an imperfect dielectric material. In tissue, heating occurs because the EM field forces water molecules in the tissue to oscillate. The bound water molecules tend to oscillate out of phase with the applied fields, so some of the EM energy is absorbed and converted to heat [24]. MWA has several theoretical advantages that may result in improved performance near blood vessels. Owing to the much broader field of power density (up to 2 cm surrounding the antenna), MWA results in a larger zone of active heating. Active RF heating occurs within several millimetres surrounding the electrode and heat distribution is primarily based on passive conduction. The increased zone in MWA allows for a more homogeneous zone of tumour cell death, both within the targeted zone and next to blood vessels. This feature is thought to make MWA less affected by heat sink, although our results contradict this assumption. We only included patients treated with the first generation MWA system employing 915 MHz. Recent developments in the field of MWA, employing higher frequency bands (2.45 GHz) or spatial energy control (thermal, field and wavelength), claim to create more predictable, larger and more spherical ablation zones [22]. Other ablation technologies include high-intensity focused ultrasound, cryoablation and laser ablation. Limited data are available concerning their efficacy and safety profile [25]. Potential disadvantages of cryoablation include cryoshock and the risk of bleeding complications due to the lack of cautery effects and coagulation of injured vessels. The specific efficacy and safety is currently being investigated [26]. In the near future, irreversible electroporation may prove to have a superior safety profile and a higher efficacy for perivascular lesions because cell death is induced using electrical energy and primarily non-thermal [27].

In the treatment of CRLM, resection is still considered the gold standard by most [28–30]. However, given the large number of studies reporting similar survival after thermal ablation for unresectable lesions, it seems conceivable to merely consider surgical resection the historical standard [5–7, 28–30]. Descriptive series comparing outcome in survival between focal therapies such as surgical resection, RFA, MWA and others are by definition eclipsed by selection bias. The issue of recurrence in the treatment with RFA has been of great importance, especially in lesions located near large vessels due to the heat-sink effect. Reported local recurrence rate ranges widely, from 2 to 60 %. In the presented study that included merely perivascular CRLM, the local control rate of 86 % advocates the use of thermal ablation for unresectable lesions, especially considering that many uncontrolled lesions were not retreated simply due to extensive recurrence elsewhere, making local (re)treatment biologically futile. The 5- and 10-year OS of 54 and 25 % for the entire group seems competitive to the reported outcomes after surgical resection and once again promotes the setup of a randomized controlled trial comparing surgical resection to thermal ablation [30]. However, in the absence of this trial, thermal ablation should still be reserved for unresectable CRLM.

This comparative multivariate analysis did not detect a difference in primary efficacy rate after 12 months nor in assisted efficacy rate for RFA versus MWA in treating perivascular and peribiliary CRLM. These results seem to conflict with the broadly adopted assumption that MWA is superior to RFA for perivascular lesions. The difference in primary efficacy rate after 3 and 12 months between RFA and MWA remains unclarified. Hypothetically, differences between the groups regarding adjuvant chemotherapy, biological aggressiveness and physiological differences in the peri-ablative inflammatory response can lead to later detection of site recurrences. However, for the RFA group the number of synchronous metastases was higher and the number of patients receiving (neo)adjuvant chemotherapy lower. Compared to RFA, MWA is a weak stimulator of local inflammation [30]. Theoretically, the greater local inflammatory response after RFA can make early diagnosis of residual or recurring disease more difficult on ^18^F–FDG– PET. Furthermore, residual vital tumour cells may have been temporary suppressed by the local IL-1- and IL-6-mediated immune response after RFA [31]. Complication rate and severity was higher for peribiliary lesions treated with MWA, although overall complication rates were low for both ablation techniques. Although the lower operator experience for the more recently introduced MWA technique could have confounded results, for both groups, the number of complications did not decrease with experience.

The study is strengthened by long-term follow-up information. Data were collected from a prospective registry that covers all metastatic colorectal cancer patients treated with thermal ablation in a high-volume single centre by two interventional radiologists with broad experience in ablation. The rationale for this strategy was the fact that MWA is nowadays promoted as superior to RFA for perivascular lesions and RFA is thought to represent a safer option for peribiliary CRLM because of the less aggressive heat production and superior ablation zone predictability. We chose primary and assisted efficacy rate as primary endpoints, because this represents a reliable and objective outcome measure for focal therapies pursuing cure. Given the superior sensitivity of intraoperative ultrasound (IOUS) to detect additional small CRLM, most lesions were treated using an open approach. Over the last decade, the accuracy of preoperative radiological staging has improved by using high-quality cross-sectional imaging techniques such as MRI with hepatospecific contrast agents and diffusion-weighted imaging. These developments may have reduced the importance of IOUS as staging technique. Nevertheless, even in centres employing state-of-the-art pre-procedural imaging, intraoperative findings still alter the course of the procedure in a considerable number of patients [32–35]. Furthermore, many patients underwent combined ablations plus resection(s) of CRLM and/or their primary tumour in a single session. Although the percutaneous approach is indisputably superior to the open approach regarding safety and invasiveness, the open approach is still thought to be superior regarding local efficacy [36, 37]. New techniques to improve visualization during percutaneous ablations, such as PET/CT-guided percutaneous ablation and US-CT/MRI image fusion, are promising [38–40]. We used ^18^F-FDG PET for follow-up in all patients, which is widely considered to represent the most sensitive technique to detect recurring disease [41].

Conclusions drawn from this retrospective series are most limited by the fact that we compared two historical cohorts with an inherent selection bias for lesions treated in the more recent era where both techniques were available. The groups were relatively small, especially given the low number of local site recurrence and complications for both groups, which enhances the possibility that our findings result from chance. The assisted efficacy rate should also be interpreted with care. Results after retreatment were assessed regardless of the type of retreatment, allowing a crossover from RFA to MWA and vice versa. However, only 6/52 recurrences were retreated using the alternate thermal ablation technique. Furthermore, the two historical cohorts obscure the use of survival as primary measure, because results may be confounded by more advanced systemic therapies. The optimal study design to assess the efficacy of the two techniques would be a prospective randomized controlled trial. Various attempts in history demonstrate the difficulties in setting-up and completing well-designed comparative studies for local therapies. For focal ablation, novel and supposedly improved methods appear with high frequency. They are introduced into general practice as part of standard care because selection of patients seems intuitive. The touted reasons are mostly theoretical and practical. Conducting randomized controlled trials has proven exceedingly difficult. As a consequence, no hard data have ever shown a clear oncological benefit of one ablation technique over the other. On the other hand, this study demonstrates that the assumption of superiority of MWA compared to RFA for perivascular lesions may have been precipitated, although the comparable outcome is reassuring. Long-term (10-year) follow-up could not be assessed for the MWA group since it was first used in our institution in 2007.

To conclude, RFA and MWA can be considered safe treatment options that appear to have equal efficacy for unresectable perivascular CRLM. Thermal ablation in the vicinity of major bile ducts seems effective although major complications can occur. Given the similar efficacy rate and lower complication rate, it is advised to use RFA instead of MWA for lesions that are located in the vicinity of the main bile ducts.
